# Supplementary material for: Real-time multimodal sensory detection using widefield hippocampal calcium imaging
Source: Commun Eng. 2023 Dec 13;2:91. doi: 10.1038/s44172-023-00144-6 (PMC10955887; doi:10.1038/s44172-023-00144-6)
Supplement: Supplementary file 2 — Supplementary Information [file 44172_2023_144_MOESM2_ESM.pdf]

Supplementary Information

Real-time Multimodal Sensory Detection Using Widefield Hippocampal Calcium Imaging

Dechuan Sun[1,2]*, Yang Yu[2], Forough Habibollahi[3], Ranjith Rajasekharan Unnithan[2], Chris French[1]*

[1]Neural Dynamics Laboratory, Department of Medicine, The University of Melbourne, Melbourne, Victoria, Australia

[2]Department of Electrical and Electronic Engineering, The University of Melbourne, Melbourne, Victoria, Australia

[3]Department of Biomedical Engineering, The University of Melbourne, Melbourne, Victoria, Australia

*** Correspondence:**
Dechuan Sun (dechuan.sun@unimelb.edu.au), Chris French (frenchc@unimelb.edu.au)

Supplementary Note 1: Decoding Model hyperparameters optimization

In this paper, our primary objective is not solely to determine the most accurate decoder, but rather to establish a functional model. Given the substantial workload involved in testing multiple models using datasets from three animals and three different experiments, we opted not to conduct a comprehensive grid search for hyperparameter optimization in certain cases. Instead of simultaneously optimizing all hyperparameters, we adopted a strategic approach. We began by identifying and tuning the most impactful hyperparameters while keeping others at reasonable values. The testing values for the grid search were determined based on previous experience and some random testing. Our hyperparameter optimization process varied for different models. For the support vector machine (SVM) model, we tested different values of the cost parameter "C". For the multilayer perceptron (MLP) model, we followed a sequential approach. We initially set the number of epochs to 50 and the unit numbers for hidden layers 1 and 2 to 150 and 30, respectively. Subsequently, we determined the optimal learning rate while maintaining fixed values for other hyperparameters. Once the learning rate was determined, we proceeded to optimize the unit numbers for hidden layers 1 and 2, followed by fine-tuning the number of epochs. For the long short-term memory (LSTM) model, we optimized hyperparameters in the following order: sliding time window size, learning rate, units for layers 1 and 2 in LSTM, and epoch number. For the convolutional neural network (CNN) model, our optimization order included learning rate, filter numbers, kernel size, pool size, and epoch number.

SVM model:

C {0.01, 0.1, 1, 10, 100}

MLP model:

learning rate {0.0001, 0.001, 0.01, 0.1}

hidden layer 1 units {150, 200, 250, 300}

hidden layer 2 units {30, 50, 150, 200}

epoch number {50, 100, 150, 200}

LSTM model:

learning rate {0.0001, 0.001, 0.01, 0.1}

Layer 1 LSTM units {32, 64, 128}

Layer 2 LSTM units {32, 64, 128}

Sliding time window size {5, 10, 15, 20}

epoch number {50, 75, 100, 150}

CNN model:

learning rate {0.0001, 0.001, 0.01, 0.1}

CNN layer 1 filters {16, 32, 64}

CNN layer 1 pool_size {(2, 1), (3, 1)}

CNN layer 1 kernel_size {(3, 1), {5,1}}

CNN layer 2 filters {16, 32, 64}

CNN layer 2 pool_size {(2, 1), (3, 1)}

CNN layer 2 kernel_size {(3, 1), {5,1}}

epoch number {50, 100, 150, 200}

Supplementary Note 2: Network architectures

Linear track:

Mouse 1: MLP

Dense layer_1 (nodes= 250, activation='relu', kernel_initializer='random_normal')

Dense layer_2 (nodes= 150, activation='relu', kernel_initializer='random_normal')

Dense layer_3 (nodes= 80, activation='softmax', kernel_initializer='random_normal')

Model (optimizer='Adam', learning_rate=0.001, loss='categorical_crossentropy', metrics=['accuracy'], epochs=100, batch_size=32)

Mouse 2: MLP

Dense layer_1 (nodes= 250, activation='relu', kernel_initializer='random_normal')

Dense layer_2 (nodes= 150, activation='relu', kernel_initializer='random_normal')

Dense layer_3 (nodes= 80, activation='softmax', kernel_initializer='random_normal')

Model (optimizer='Adam', learning_rate=0.001, loss='categorical_crossentropy', metrics=['accuracy'], epochs=100, batch_size=32)

Mouse 3: SVM

SVC(C=10, gamma=1/N)), where N is the reciprocal of the number of input features.

Visual stimuli experiment:

Mouse 1: SVM

SVC(C=1, gamma=1/N)), where N is the reciprocal of the number of input features.

Mouse 2: SVM

SVC(C=1, gamma=1/N)), where N is the reciprocal of the number of input features.

Mouse 3: MLP

Dense layer_1 (nodes= 250, activation='relu', kernel_initializer='random_normal')

Dense layer_2 (nodes= 150, activation='relu', kernel_initializer='random_normal')

Dense layer_3 (nodes= 80, activation='softmax', kernel_initializer='random_normal')

Model (optimizer='Adam', learning_rate=0.001, loss='categorical_crossentropy', metrics=['accuracy'], epochs=100, batch_size=32)

Auditory stimuli experiment:

Mouse 1: CNN

Input(shape = input_shape)

Conv2D(filters = 32, kernel_size=(3, 1), activation="relu", padding='same')

MaxPooling2D (pool_size=(2, 1))

Conv2D(filters = 32, kernel_size=(3, 1), activation="relu", padding='same')

MaxPooling2D (pool_size=(2, 1))

Flatten()

Dropout(0.2)

Dense(3, activation="softmax")

Model (optimizer='Adam', learning_rate=0.001, loss='categorical_crossentropy', metrics=['accuracy'], epochs=100, batch_size=32)

Mouse 2: CNN

Input(shape = input_shape)

Conv2D(filters = 64, kernel_size=(3, 1), activation="relu", padding='same')

MaxPooling2D (pool_size=(2, 1))

Conv2D(filters = 64, kernel_size=(3, 1), activation="relu", padding='same')

MaxPooling2D (pool_size=(2, 1))

Flatten()

Dropout(0.2)

Dense(3, activation="softmax")

Model (optimizer='Adam', learning_rate=0.001, loss='categorical_crossentropy', metrics=['accuracy'], epochs=150, batch_size=32)

Mouse 3: CNN

Input(shape = input_shape)

Conv2D(filters = 32, kernel_size=(5, 1), activation="relu", padding='same')

MaxPooling2D (pool_size=(3, 1))

Conv2D(filters = 64, kernel_size=(5, 1), activation="relu", padding='same')

MaxPooling2D (pool_size=(3, 1))

Flatten()

Dropout(0.2)

Dense(3, activation="softmax")

Model (optimizer='Adam', learning_rate=0.001, loss='categorical_crossentropy', metrics=['accuracy'], epochs=100, batch_size=32)

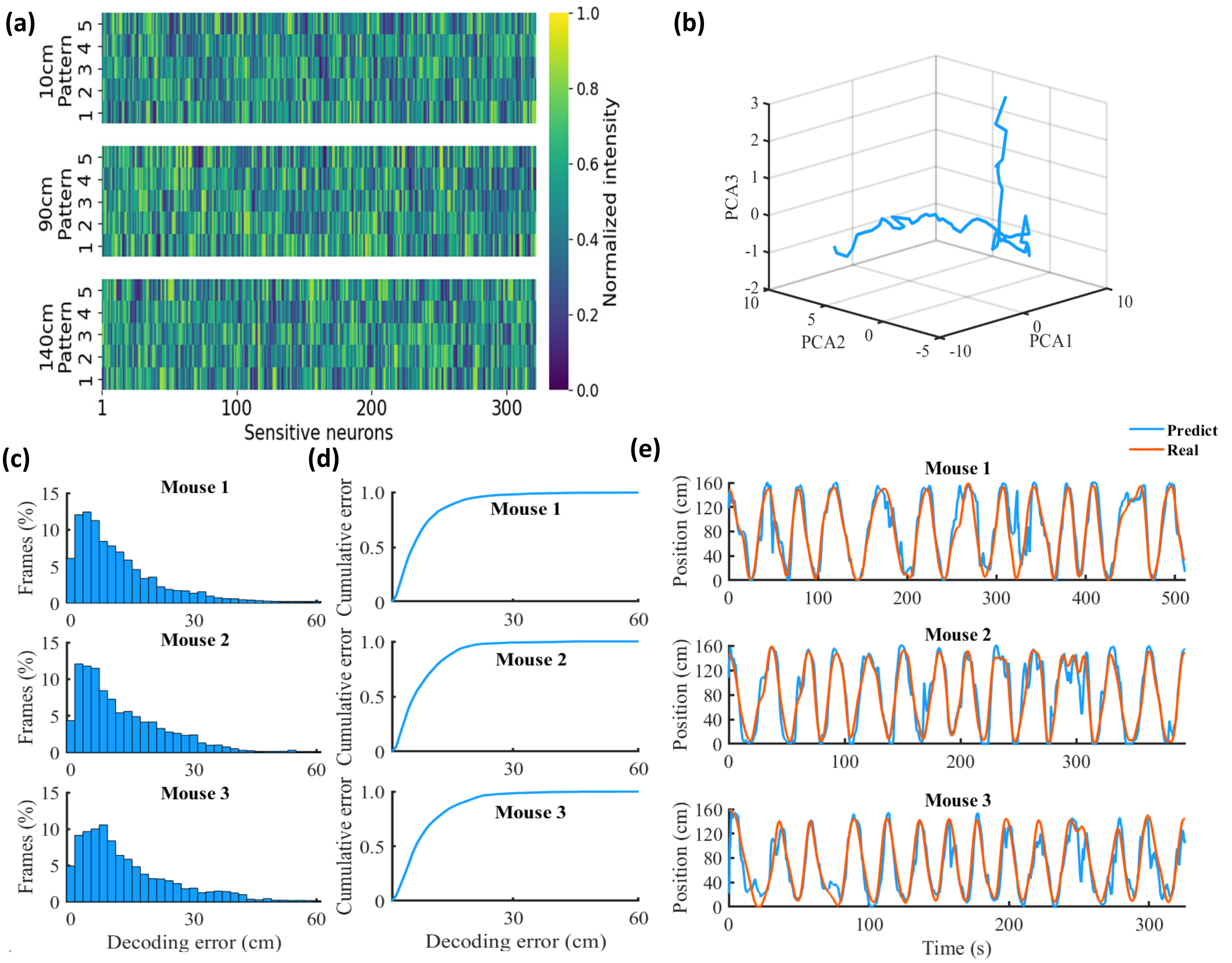

Supplementary Figure S1. Position reconstruction experiment. (a) Examples of the firing patterns of sensitive neurons in linear track experiments. **(b)** The neuronal ensemble activity of a mouse running in a linear track described in a high-dimensional neural state space. Features were extracted from the output of the last hidden layer in a multilayer perceptron neural network model. Principle component analysis (PCA) was applied to features for better visualization**. (c, d)** Histogram and the cumulative fraction of the decoding error in the real-time session. The decoding model with the best performance in the training session was implemented in each mouse respectively. **(e)** The running trajectory reconstruction of a mouse in the real-time session. The red curve demonstrates the real position of a mouse tracked by the video camera and the blue curve represents the reconstructed position.

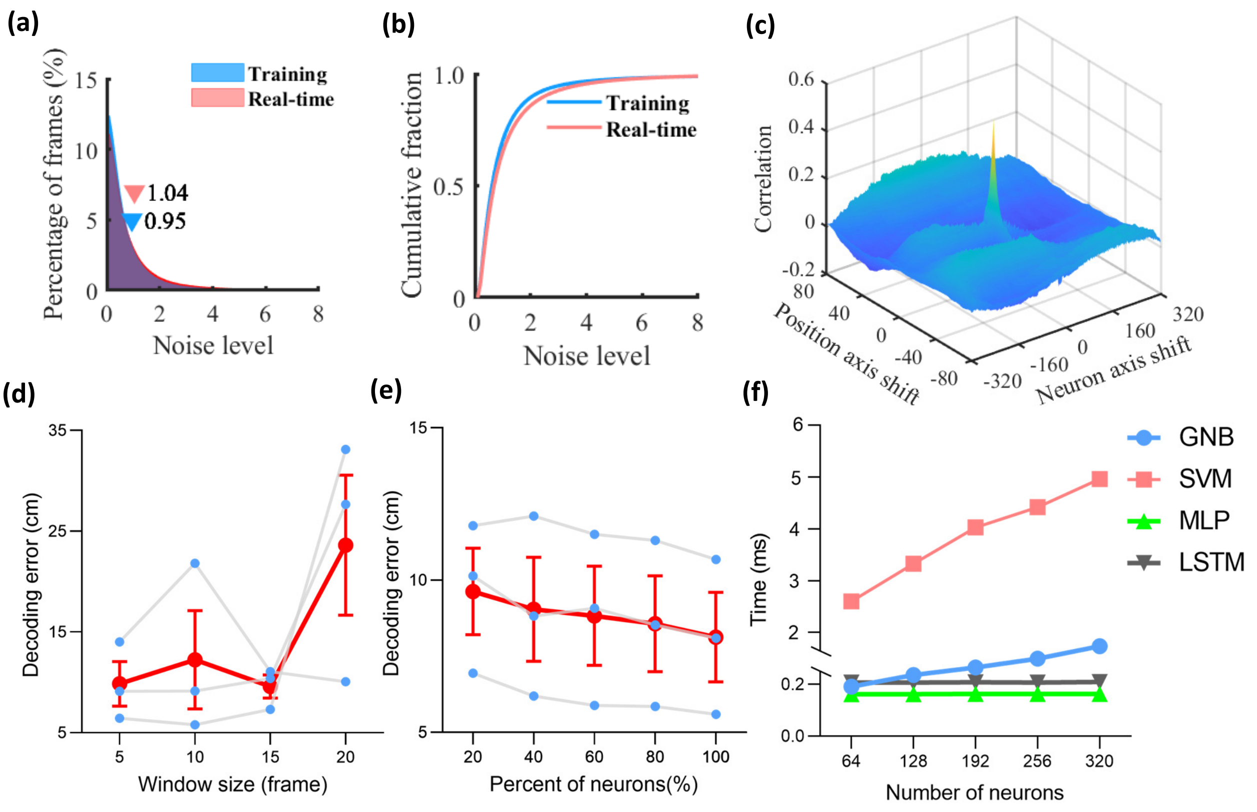

Supplementary Figure S2. Position reconstruction decoding models. (a, b) Histogram and the cumulative fraction of the noise level in the training session and real-time session (paired Student's t-test did not show significant differences: t=0.04121, df=79, p-value = 0.9672). **(c)** Cross-correlation of place field maps between the training session and real-time session. **(d)** Position reconstruction error as a function of the time window size using the long short-term memory model. Blue dots represent the decoding error measured in three mice, and the red dots represent the mean error (Pearson correlation, R^2= 0.5646, p-value = 0.2486). **(e)** Position reconstruction error as a function of the percentage of sampled neurons using a multilayer perceptron model. Error decreased by incorporating more neurons (Pearson correlation, R^2= 0.9716, p-value = 0.002). **(f)** Processing time as a function of the number of sampled neurons in a mouse using different models (Pearson correlation, Gaussian naïve Bayes (GNB): R^2= 0.9926, p-value = 0.0003; support vector machine (SVM): R^2= 0.9876, p-value = 0.0006; multilayer perceptron (MLP): R^2= 0.7701, p-value = 0.0505; long short-term memory (LSTM): R^2= 0.5103, p-value = 0.1752).

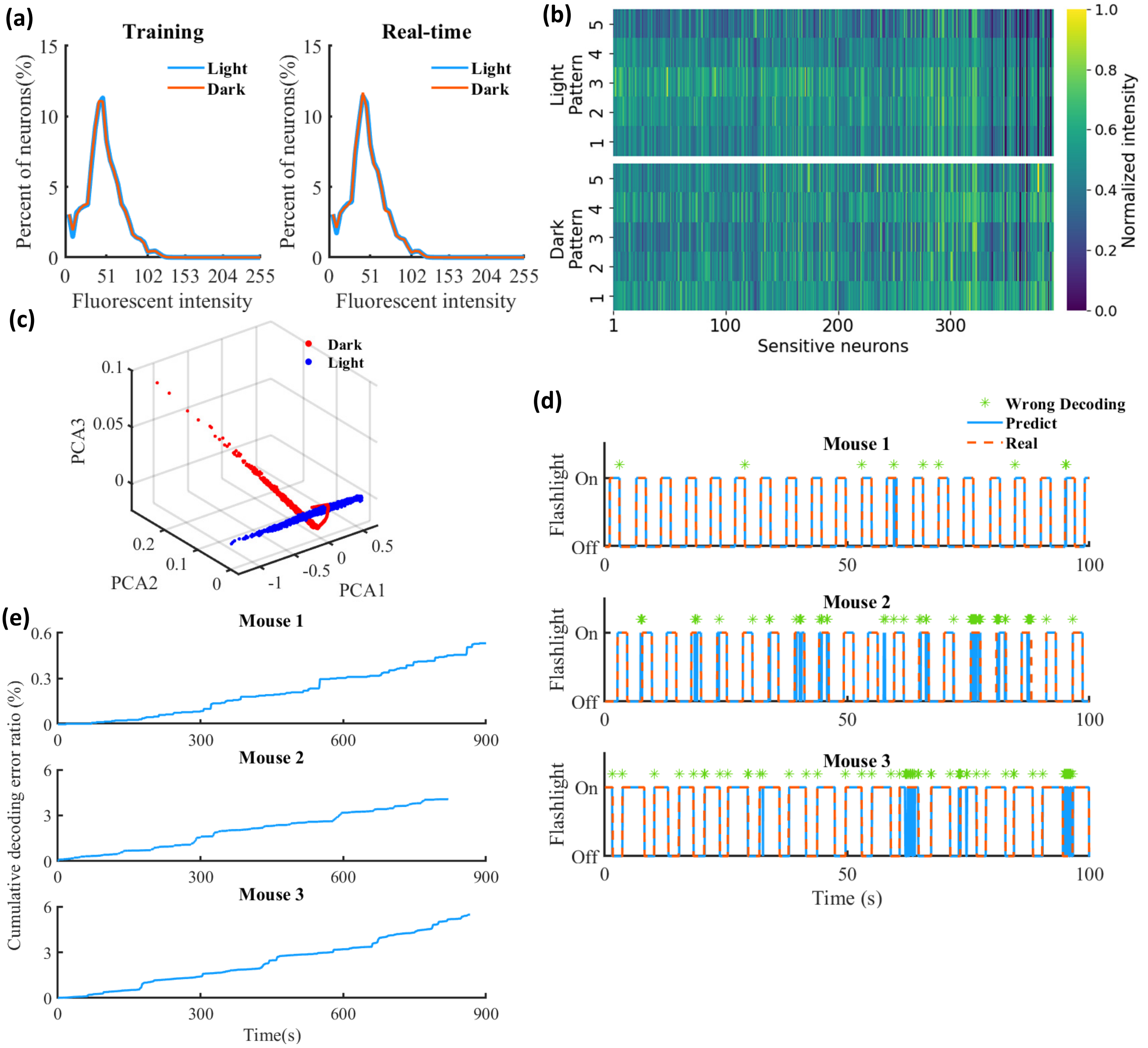

Supplementary Figure S3. Visual stimuli identification experiment. (a) Distributions of neurons' fluorescent intensity. Paired Student's t-test didn't show significant differences (Training: t= 4.403×10^{-14}, df=51, p-value > 0.9999; Real-time: t= 9.703×10^{-14}, df=51, p-value > 0.9999). **(b)** Examples of the firing patterns of sensitive neurons in visual stimuli experiments. **(c)** Visual stimuli responses described in a high-dimensional neural state space. Features were extracted from the output of the last hidden layer in a multilayer perceptron neural network model. Principle component analysis (PCA) was applied to features for better visualization. **(d)** Examples of the decoding performance in the real-time session (100×30 frames). The decoder with the best performance in the training session was used in each mouse respectively. The red dashed line and solid blue line represent the real and predicted status of the auditory stimuli respectively. The wrong decoding frame is marked with a green star. **(e)** Cumulative decoding error ratio with respect to time.

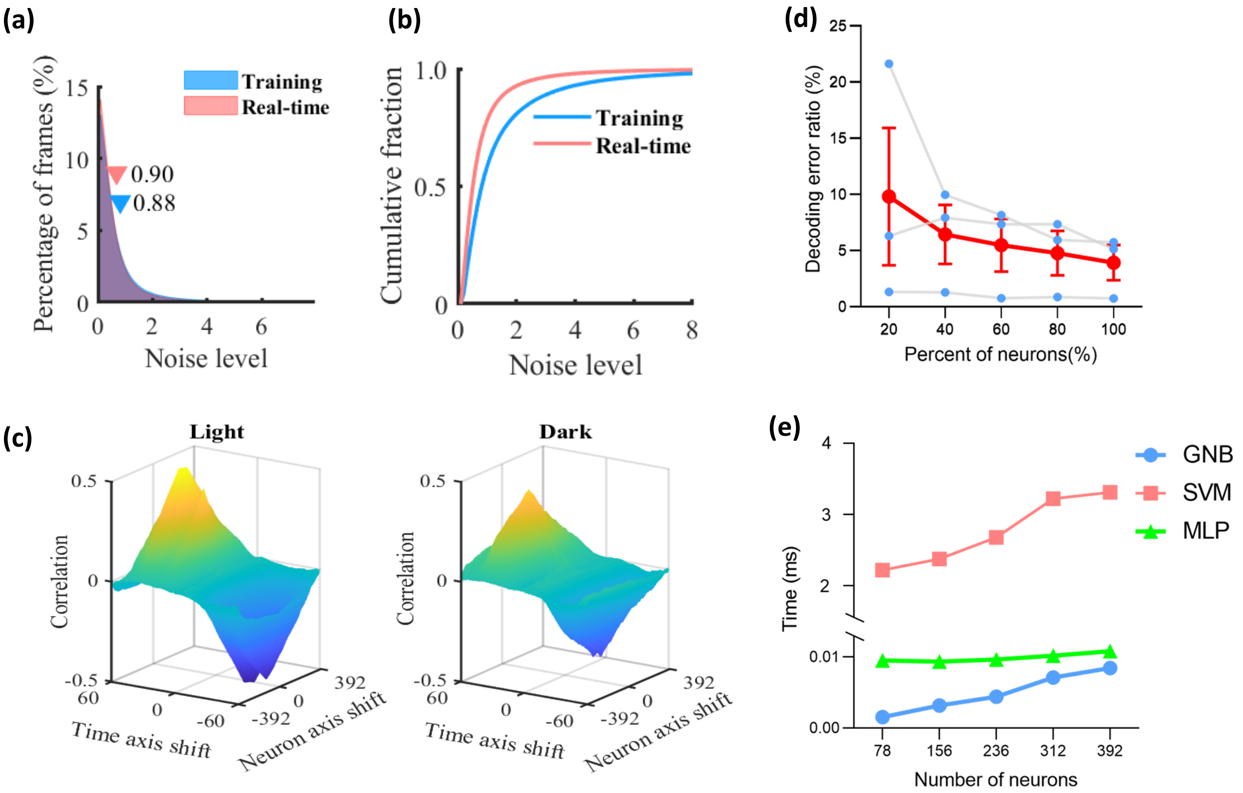

Supplementary Figure S4. Visual stimuli decoding models. (a, b) Histogram and the cumulative fraction of the noise level in the training session and real-time session (paired Student's t-test did not show significant differences: t=0.1362, df=79, p-value = 0.8920). **(c)** Cross-correlation of firing rate maps between the training session and real-time session in light and dark environments. The unit of time axis shift is the frame, which is around 33ms. **(d)** Decoding error as a function of the percentage of sampled neurons using a multilayer perceptron model. Blue dots represent the decoding error measured on three mice, and the red dots represent the mean error. Error decreased considering more neurons (Pearson correlation, R^2= 0.8702, p-value = 0.0207). **(e)** Processing time as a function of the number of sampled neurons in a mouse using different models (Pearson correlation, Gaussian naïve Bayes (GNB): R^2= 0.9853, p-value = 0.0008; support vector machine (SVM): R^2= 0.9523, p-value = 0.0045; multilayer perceptron (MLP): R^2= 0.8162, p-value = 0.0355).

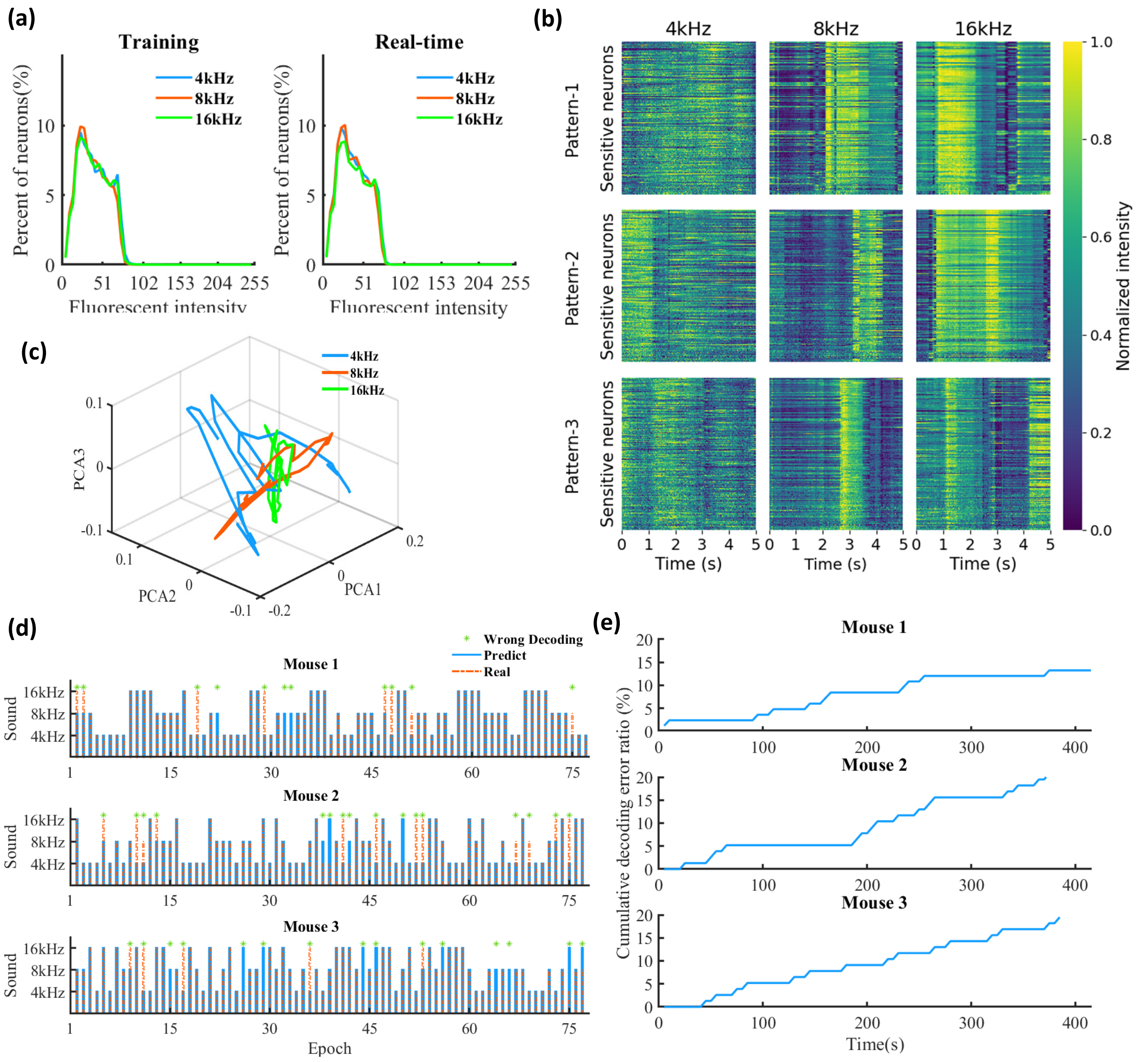

Supplementary Figure S5. Auditory stimuli identification experiment. (a) Distributions of neuronal fluorescent intensity. One-way analysis of variance did not show significant differences (Training: $F_{(2,102)}=0.4342$, p-value = 0.6490; Real-time: $F_{(2,102)}=2.853$, p-value = 0.0623). **(b)** Examples of the firing patterns of sensitive neurons in auditory stimuli experiments. **(c)** Auditory stimuli responses described in a high-dimensional neural state space. Features were extracted from the output of the last hidden layer in a convolutional neural network (CNN) model. Principle component analysis (PCA) was applied to features for better visualization. **(d)** Examples of the decoding performance using the CNN model in the real-time session. The red dashed line and solid blue line represent the real and predicted status of the auditory stimuli respectively. The wrong decoding frame is marked with a green star. **(e)** decoding error ratio with respect to time.

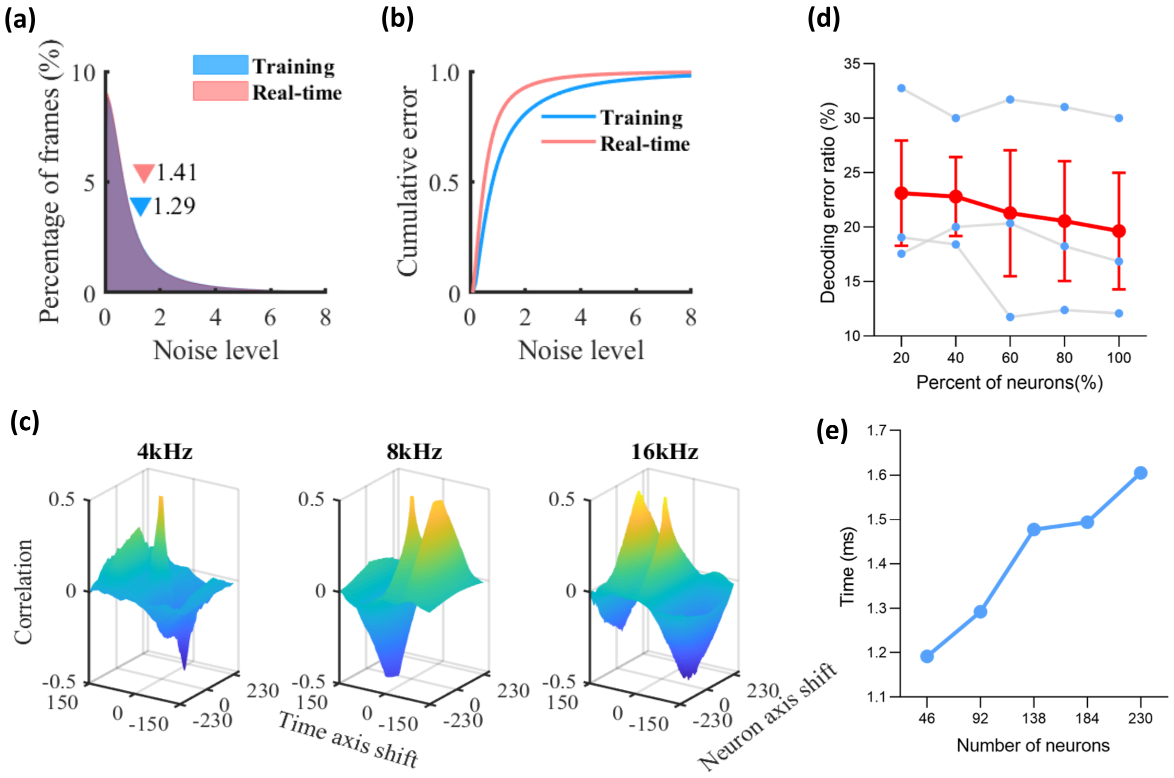

Supplementary Figure S6. Auditory stimuli decoding models. (a, b) Histogram and the cumulative fraction of the noise level in the training session and real-time session (paired Student's t-test did not show significant differences: t=1.226, df=79, p-value = 0.2238). **(c)** Cross-correlation of firing rate maps between the training session and real-time session in three environments. The unit of time axis shift is the frame, which is around 33ms. **(d)** Decoding error as a function of the percentage of sampled neurons using the convolutional neural network (CNN) model. Blue dots represent the decoding error measured on three mice, and the red dots represent the mean error. Error decreased considering more neurons (Pearson correlation, R^2= 0.9717, p-value = 0.002). **(e)** Processing time as a function of the number of sampled neurons in a mouse using the CNN model (Pearson correlation, Gaussian naïve Bayes (GNB): R^2= 0.9518, p-value = 0.0046).

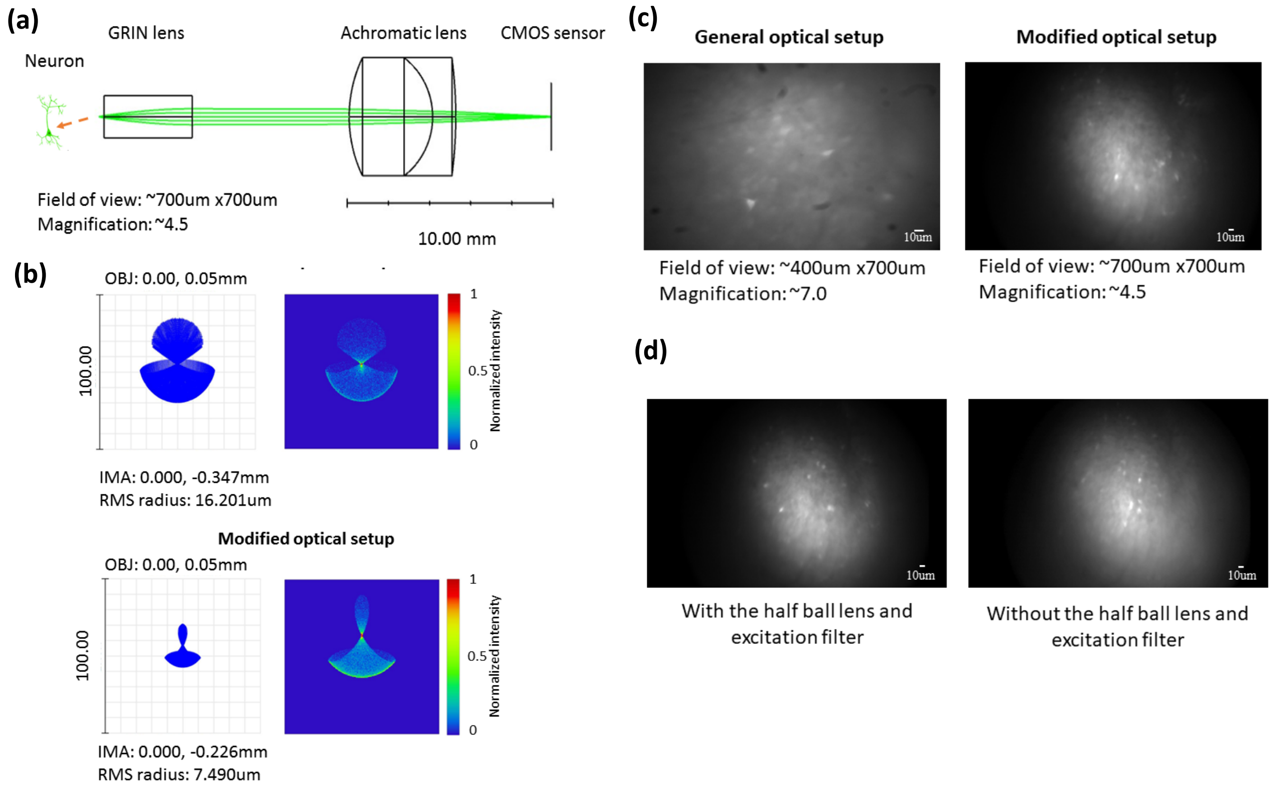

Supplementary Figure S7. Simulation and imaging of hippocampal CA1 neurons in freely behaving mice. **(a)** Zemax simulation of emission path of our modified optical system. **(b)** The spot diagram and normalized intensity distribution are shown for an object located at (0.00mm, 0.05mm). **(c)** The modified optical system has a smaller magnification in comparison to the conventional one and provides a larger field of view. **(d)** Imaging with/without a half-ball lens and an excitation filter shows similar imaging quality.

Decoder ╱ mean / median	Mouse_1	Mouse_2	Mouse_3	All (cm/frame)
**GNB**	17.76<br>12	20.00<br>14	30.60<br>22	22.79 ± 3.42<br>16.00 ± 2.64
**SVM**	10.45<br>8	16.42<br>12	13.06<br>10	13.31 ± 1.49<br>10.00 ± 1.00
**MLP**	10.29<br>8	16.24<br>12	16.98<br>14	14.50 ± 1.83<br>11.33 ± 1.52
**LSTM**	11.16<br>10	17.13<br>12	16.44<br>14	14.91 ± 1.63<br>12.00 ± 1.00

Supplementary Table 1. The mean and median decoding error (cm/frame) in the training session of the position reconstruction experiment using Gaussian naïve Bayes (GNB), support vector machine (SVM), multilayer perceptron (MLP), and long short-term memory (LSTM) decoders. The first row in each cell represents the mean error and the second row represents the median error. The overall error is expressed as mean/median ± standard error mean. Significant differences in average decoding error were observed among different decoders (One-way analysis of variance, $F_{(3,6)} = 5.674$, p-value = 0.0347). Post-hoc tests using Fisher's least significant difference revealed that the average decoding error using the GNB decoder was significantly higher than that using the SVM, MLP, and LSTM decoders, with a p-value of 0.0102, 0.0180, and 0.0220, respectively. No significant differences were found between the other groups: SVM-MLP (p-value = 0.6586), SVM-LSTM (p-value = 0.5563), and MLP-LSTM (p-value = 0.8794). Significant differences in median decoding errors were detected among different decoders (One-way analysis of variance, $F_{(3,6)}=4.000$, p-value = 0.0701). Post-hoc analysis using Fisher's least significant difference test showed that the median decoding error using the GNB decoder was significantly higher than that using SVM (p-value = 0.0167) and MLP (p-value = 0.0431). No significant differences were found between the other pairs: GNB-LSTM (p-value = 0.0710), SVM-MLP (p-value = 0.4927), SVM-LSTM (p-value = 0.3153), and MLP-LSTM (p-value = 0.7275).

Decoder	Mouse_1	Mouse_2	Mouse_3	All
**GNB**	22.95%	34.28%	4.40%	20.54% ± 8.70%
**SVM**	3.99%	4.01%	1.22%	3.08% ± 0.92%
**MLP**	5.36%	4.63%	0.73%	3.57% ± 1.43%

Supplementary Table 2. The average decoding error ratio in the training session of the light stimuli experiment using Gaussian naïve Bayes (GNB), support vector machine (SVM), and multilayer perceptron (MLP) decoders. The overall error ratio is expressed as mean ± standard error mean. Significant differences in average decoding error were observed among different decoders (One-way analysis of variance, $F_{(2,4)}=17.39$, p-value = 0.0106). Post-hoc analysis using Fisher's least significant difference test revealed that the average decoding error with the GNB decoder was significantly higher than that with SVM (p-value = 0.0067) and MLP (p-value = 0.0073). However, no significant differences were found between SVM and MLP decoders (p-value = 0.9071).

Decoder	Mouse_1	Mouse_2	Mouse_3	All
**GNB**	71.09%	77.50%	81.34%	76.64% ± 2.59%
**SVM**	42.81%	39.85%	40.05%	40.91% ± 0.82%
**MLP**	39.33%	37.16%	37.84%	38.11% ± 0.55%
**CNN**	17.67%	22.76%	22.89%	21.11% ± 1.48%

Supplementary Table 3. The average decoding error ratio in the training session of the sound stimuli experiment using Gaussian naïve Bayes (GNB), support vector machine (SVM), multilayer perceptron (MLP), and convolutional neural network (CNN) decoders. The overall error ratio is expressed as mean ± standard error mean. Significant differences in average decoding error were observed among different decoders (One-way analysis of variance, $F_{(3,6)}$=154.5, p-value < 0.0001). Fisher's least significant difference post-hoc test revealed that the average decoding error using the GNB decoder was significantly higher than that using SVM, MLP, and CNN decoders (p-value < 0.0001). However, no significant differences were found between SVM and MLP decoders (p-value = 0.3333). Additionally, decoding accuracy using CNN decoder was significantly higher than that using SVM and MLP decoders, with a p-value of 0.0003 and 0.0007, respectively.
